# An improved BM25 algorithm for clinical decision support in Precision Medicine based on co-word analysis and Cuckoo Search

**DOI:** 10.1186/s12911-021-01454-5

**Published:** 2021-03-02

**Authors:** Zicheng Zhang

**Affiliations:** 1 School of Information Management, Nanjing University, Nanjing, 210023 China; 2 Jiangsu Key Laboratory of Data Engineering and Knowledge Service, Nanjing, 210023 China

**Keywords:** Clinical decision support, Precision Medicine, Information retrieval, Co-word analysis, Improved BM25, Cuckoo Search

## Abstract

**Background:**

Retrieving gene and disease information from a vast collection of biomedical abstracts to provide doctors with clinical decision support is one of the important research directions of Precision Medicine.

**Method:**

We propose a novel article retrieval method based on expanded word and co-word analyses, also conducting Cuckoo Search to optimize parameters of the retrieval function. The main goal is to retrieve the abstracts of biomedical articles that refer to treatments. The methods mentioned in this manuscript adopt the BM25 algorithm to calculate the score of abstracts. We, however, propose an improved version of BM25 that computes the scores of expanded words and co-word leading to a composite retrieval function, which is then optimized using the Cuckoo Search. The proposed method aims to find both disease and gene information in the abstract of the same biomedical article. This is to achieve higher relevance and hence score of articles. Besides, we investigate the influence of different parameters on the retrieval algorithm and summarize how they meet various retrieval needs.

**Results:**

The data used in this manuscript is sourced from medical articles presented in Text Retrieval Conference (TREC): Clinical Decision Support (CDS) Tracks of 2017, 2018, and 2019 in Precision Medicine. A total of 120 topics are tested. Three indicators are employed for the comparison of utilized methods, which are selected among the ones based only on the BM25 algorithm and its improved version to conduct comparable experiments. The results showed that the proposed algorithm achieves better results.

**Conclusion:**

The proposed method, an improved version of the BM25 algorithm, utilizes both co-word implementation and Cuckoo Search, which has been verified achieving better results on a large number of experimental sets. Besides, a relatively simple query expansion method is implemented in this manuscript. Future research will focus on ontology and semantic networks to expand the query vocabulary.

## Background

With the proliferation of computer technologies, the information available on the Internet has swiftly increased leading to various implementations utilized for information extraction from medical articles. Hence, medical treatment techniques have stepped into the age of Big Data. However, managing the immense data and extracting information from them is a critical endeavor. If this process can be improved, the advantages that it could offer would be so beneficial for medical doctors. For instance, some routine decision-making tasks require significant repetition, which takes time and increases costs. However, computerized medical information retrieval systems can effectively improve efficiency, save costs, and reduce errors. Proper use of computer technology can bring efficacy to all fields where it will be used. Therefore, the development of medical information retrieval systems is crucial. In reality, every decision of a doctor is critical to the patient, so the doctor must follow the state-of-the-art techniques and keep abreast with the latest technology and methods of clinical science. The academic literature providing the latest research results in the medical community can be accessed via the Internet and the medical retrieval models play a crucial role. Furthermore, searching the relevant biomedical literature on the Internet for a reference can be highly beneficial for medical practitioners who encounter a difficult problem on a certain medical record.

Information Retrieval (IR) methods for Clinical Decision Support (CDS) have been the focus of recent research and assessment campaigns. Specifically, the CDS track between 2014 and 2016 Text Retrieval Conferences (TREC) [1–3] sought to assess the systems providing evidence-based information in the form of either full-text or abstracts from an open-access subset of MEDLINE to the clinicians in return to their queries. Furthermore, the tracks from 2017 to 2019 [4–6] focused on important implementations in clinical decision support providing both useful and precise medical information to clinicians treating cancer patients. In these, each case described the disease (a type of cancer), the relevant genetic variants (which genes), and basic demographic information (age and sex) of patients. Precision Medicine introduced in [7] is a new medical concept utilizing individualized medicine that develops with the rapid progress of genome sequencing technology and the cross-application of bioinformatics and Big Data science.

## Preleminaries

The IR aims to retrieve related documents based on a given query. The relevancy of documents to queries is often gauged by the score assigned by an IR model, e.g., the widely-implemented BM25 model [8]. On the one hand, the past few decades witnessed the implementation of machine learning technology when information retrieval was a concern. The document ranking process could be classified into three groups as follows: (i) the single document methods, (ii) the document pair methods, and (iii) the document list methods. The common single-document methods, such as [9] utilizing a logistic regression technique, deal with a feature vector of each document as an input, where the output is the relevance of each document. The document pair methods, e.g., the ones utilizing Rank-SVM [10] or Rank-Boost [11], implement a feature vector of a pair of documents as the input and use the correlation between the documents as the output. The document list methods, e.g., the ones proposed List-Net [12], Ada-Rank [13], or Lambda-Mart [14], employ a set of documents associated with a query as the input and a ranked list as the output. In recent years, query expansion methods have been widely implemented in information retrieval. Singh et al. [15] suggested a method based on fuzzy logic, in which the top-ranked documents were regarded as relevant feedback documents for mining query information. Furthermore, the choice of different query expansion terms was determined according to their importance. These methods often assign each term to a different relevance score and then select the expansion term based on a certain threshold.

Keikha et al. [16] considered the Wikipedia corpus as the feedback set space to train the Word Vector Model and determined the long-term selection of the best features in both supervised and unsupervised models. Almasri et al. [17] also utilized vectors to represent query words and query expansion terms returned by pseudo-correlation feedback. They added cosine similarity is to the Bag-of-Words Model, and the frequency of each word in the query term was recalculated. Singh et al. [18] proposed a classic correlation feedback method, which increased the entry weight of the related documents and reduced it to that of the non-relevant ones. However, one of the disadvantages of this method was to be very time-consuming for practitioners in assessing the relevance of documents.

Cui et al. [19] developed a query expansion method for web search logs utilizing the interaction information of practitioners. The key assumption behind this method was that the documents chosen by a user to read were related to the query. The new words in the related documents were sorted according to their similarity with the user query, and the new words with the highest similarity were selected as the expanded word. The candidate expanded words were extracted from the top documents, and then the candidate expanded words were weighted and sorted by the probability generated by the language model. Aronson and Rindflesch [20] proposed a method based on the Unified Medical Language System (UMLS) query expansion, which benefitted from the Meta-Map program [21] to identify the medical phrases in the original query and then expanded the query with new phrases. Hence, the experimental results showed that the query expansion utilizing the UMLS was an effective method to improve the performance of information retrieval.

Li et al. [22] proposed a method of keyword-weighted network analysis to implement a medical full-text recommendation, which helped to expand the medical acronym list by searching the full-text. Domain experts verified that the algorithm worked well in terms of accuracy in recommending medical literature. Balaneshinkordan et al. [23] developed a query expansion method utilizing the Bayesian approach, which expanded the genes of a disease to be no less than three words. The experiments revealed that the algorithm had a higher precision value.

The literature review brings us the idea of using both query expansion and keywords to retrieve documents that are highly related to a query. Hence, this manuscript proposes a method utilizing expanded words and co-word analysis as new tools to optimize the information retrieval of biomedical articles implementing the BM25 algorithm as a base method. This is to compute scores of the abstract, expanded words, and co-word as a composite retrieval function. Besides, when a disease and a gene both appear in the same biomedical article, the score of the article tends to increase. Finally, the Cuckoo Algorithm [28] is utilized to optimize the parameters of the proposed retrieval algorithm.

As a classical information retrieval algorithm, BM25 has been frequently implemented on TREC, such as 2017, 2018, and 2019 Precision Medicine [34–49]. These algorithms mainly utilize either the original BM25 algorithm or its improved version to retrieve information [37, 38].

## Experimental data

### Data structure

**Fig. 1:**
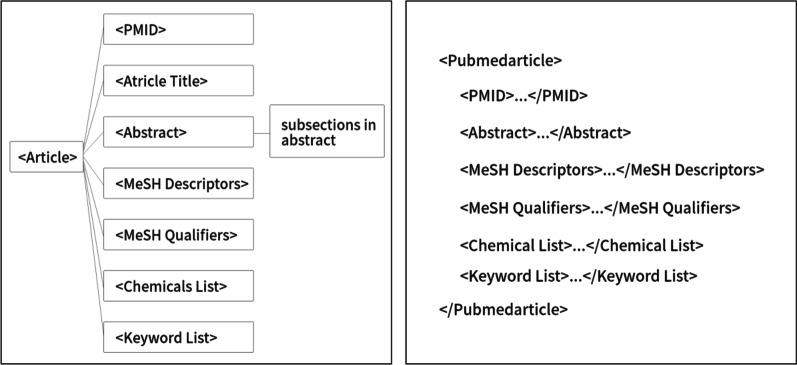
General structure and the XML attributes of MEDLINE abstracts

The abstracts of biomedical articles are presented in XML format. The MeSH headings, chemical lists, and keyword lists for XML documents are selected to utilize abstracts whose displays are presented in Fig. 1.

### Data distribution

While the total number of biomedical articles in both 2017 and 2018 TREC Precision Medicine is 26,613,834, the 2019 set has 29,137,637 articles. Table 1 shows some of the statistics that are used in information retrieval, where Abstract-Mean-Length represents the average length of the abstracts after deleting stop-words; Abstract-Number represents the number of articles with abstracts; Chemical-Mean-Length represents the average length of the chemical lists; Chemical-Number represents the number of articles with a chemical list; Mesh-Mean-Length represents the average length of the MeSH headings; Mesh-Number represents the number of articles with MeSH headings; Keyword-Mean-Length represents the average length of the keyword list, and Keyword-Number represents the number of articles with keyword lists.

**Table 1:** The statistics of the TREC Precision Medicine covering the period of 2017–2019

Name	2017 and 2018	2019
Abstract-Mean-Length	77.5	83.5
Abstract-Number	26,613,834	29,137,637
Chemical-Mean-Length	3.8	3.8
Chemical-Number	13,113,093	13,670,358
Mesh-Mean-Length	10.5	10.6
Mesh-Number	24,387,151	25,389,659
Keyword-Mean-Length	4.1	4.4
Keyword-Number	4,005,446	5,435,471

### Query expansion

Medical Subject Headings (MeSH) is a controlled vocabulary developed by the U.S. National Library of Medicine, which is mainly utilized to index, catalog, and search articles relevant to both biomedical and health sciences. The important role of the MeSH in medical information retrieval is mainly manifested with two aspects, namely accuracy and specificity. While indexers input information into the retrieval system, researchers utilize this information concerning the two aspects. The MeSH is used as the platform making the terms consistent between the index and search to achieve the best outcomes. Hence, the accurate and comprehensive usage of the MeSH has a significant impact on the results of information retrieval. In this manuscript, we utilize the MeSH database (meshb.nlm.nih.gov/MeSHonDemand) to find expansion terms or new words and their broader terms. The MeSH on Demand is utilized to expand query terms and obtain additional terms if possible.

Table 2 shows Topic 2017-1 as an example, and Table 3 presents the results of the extended words.

**Table 2:** The retrieval topic description of the TREC Precision Medicine

Year	Disease	Gene	Demographic characteristics	Other
2017–1	Liposarcoma	CDK4 Amplification	38-year-old male	GERD
2018–1	Melanoma	BRAF (V600E)	64-year-old male	None
2019–1	Melanoma	BRAF (E586K)	64-year-old female	None

**Table 3:** The expanded MeSH of 2017 TREC Precision Medicine retrieval task 1

Search word	Expanded word
Liposarcoma	Myxoid
CDK4 Amplification	Cyclin-Dependent Kinase 4
	Proto-Onkogene Proteins c-mdm2
38-year-old	Middle Aged
	Adult
Male	Human

### Age expansion

The variable age included in the demographic field is expanded to the terms or new words proposed by Kastner et al. [24]. We have readjusted the age division and assumed that those over 18 should be adults. Table 4 presents our expansion model that is based on variable age.

**Table 4:** The expanded age of the TREC Precision Medicine

Term	Range
Fetus	Fetus
Newborn	Birth to 1 month
Infant	> 1 month to < 24 months
Preschool	2 years to < 6 years
Child	6 years to < 13 years
Adolescent	13 years to < 19 years
Young	19 years to < 35 years
Middle age	35 years to < 60 years
Aged	60 years to < 80 years
Aged 80	≥ 80 years
Adult	≥ 18 years

## The proposed model

We first utilize the MeSH on Demand to find MeSH terms and additional terms that can be used in the retrieval of abstracts for any given query. Then, we construct a “wordlist” including chemical words, keywords, and MeSH headings that utilizes query expansion, thereby finding documents that are more related to query expansion. This is to increase the relevance score of documents. In the next step, we performed the co-word analysis utilizing either each separate resource, such as abstract, keywords, chemical words, or MeSh headings, or all sources at a time to find co-occurrence of selected words, such as disease and gene, in our case. While the first step deals with computing the score of abstracts based on a query and its morpheme, the second step deals with calculating the score of expansion words. Then, the score of the co-word is calculated. Hence, as long as the number of documents is reduced based on the “word list”, the score of the co-word tend to increase. In the last step, we compute the composite score consisting of three scores of abstract, expanded words, and co-word. Afterward, Cuckoo Search [28], an evolutionary optimization method, is applied to optimize the parameters of the proposed retrieval model.

### The abstract scoring model

The BM25 [8] is a classical information retrieval model that is based on analyzing a query $Q$ to find a morpheme $qi$. For each search result $d$, it calculates the correlation between each morpheme $qi$ and $d$, and finally gives a weight to the sum of the correlation score of $qi$ concerning $d$ to obtain a correlation score between $Q$ and $d$. The general formula of the BM25 can be expressed by:

1 $$Score\left( {Q,d} \right) = \mathop \sum \limits_{i}^{n} W_{i} \times R\left( {q_{i} ,d} \right)$$

where $W_{i}$ is a weight determining the relevance of a word to a document.

The Inverse Document Frequency (IDF) is defined as:

2 $$IDF\left( {q_{i} } \right) = log\frac{D}{{card(\{ q_{i} |i \in d_{i} \} )}}$$

where $D$ represents the total number of corpus documents, and $card(\{ j|i \in d_{i} \} )$ represents the number of documents containing morpheme $qi$. According to (2), the more $qi$ contained in a document, the lower the weight of $qi$ for a given set of documents. In other words, when several documents contain $qi$, the discrimination of $qi$ is not so robust that the importance of utilizing $q_{i}$ to judge relevance is so weak.

The relevance score $R \left( {q_{i} , d} \right)$ of morpheme $q_{i}$ documenting $d$ is defined as:

3 $$R\left( {q_{i} ,d} \right) = \frac{{f_{i} \times \left( {k_{1} + 1} \right)}}{{f_{i} + K}} \times \frac{{qf_{i} \times \left( {k_{2} + 1} \right)}}{{qf_{i} + k_{2} }}$$

where parameter $K$ is:

4 $$K = k_{1} \times \left( {1 - b_{1} + b_{1} \times \frac{dl}{{avgdl}}} \right)$$

where, $k_{1}$, $k_{2}$, and $b$ are adjustment factors that are usually set according to experience, $f_{i}$ is the frequency of $q_{i}$ in $d$, $qf_{i}$ is the frequency of $q_{i}$ in query, $dl$ is the length of document $d$, and $avgdl$ is the average length of all documents. In most cases, $q_{i}$ appears only once in the query, i.e., $qf_{i}$ = 1. Hence, (3) can be rewritten as:

5 $$R\left( {q_{i} ,d} \right) = \frac{{f_{i} \times \left( {k_{1} + 1} \right)}}{{f_{i} + K}}$$

As seen from the definition, the role of parameter $b$ is to tune the impact of the document length on the relevance. The larger the $b$ is, the greater the impact of the document length on the relevance score will be, and vice versa. Similarly, the longer the relative length of the document is, the larger the $K$, and hence the smaller the relevance score will be. In the end, the correlation score of the abstract of the document $d$ can be expressed as:

6 $$Score_{abstract} \left( {Q,d} \right) = \mathop \sum \limits_{i}^{n} IDF\left( {q_{i} } \right) \times \frac{{f_{i} \times \left( {k_{1} + 1} \right)}}{{f_{i} + k_{1} \times \left( {1 - b_{1} + b_{1} \times \frac{dl}{{avgdl}}} \right)}}$$

### The expanded word score

As seen in Table 1, most biomedical articles have both abstracts and titles. The number of biomedical articles containing chemical words, MeSH headings, and keywords varies widely. Specifically, there exist 13,113,093 articles containing chemical words, 2,438,717,151 articles containing MeSH headings, and 4,005,446 articles containing keywords. As the literature suggests, direct utilization of the BM25 leads to failure when dealing with a large selection of documents [50]. In this subsection, we propose an improved BM25 algorithm to compute the scores of expanded words. We combine chemical words, MeSH headings, and keywords into a list called ‘Word List’. The length of the “Word List” in the document is defined by:

7 $$dwl = dcl + dml + dkl$$

where $dcl$ is the length of chemical words in document $d$, $dml$ is the length of MeSH headings in document $d$, and $dkl$ is the length of keywords in document $d$.

The IDF value of the expanded word appearing in the “Word List” of document $d$ can be given by:

8 $$IDF_{word} \left( {q_{i} ,d} \right) = log\frac{{N - n\left( {q_{i} } \right) + 0.5}}{{n\left( {q_{i} } \right) + 0.5}}$$

where $N$ represents the number of documents in which $dwl$ > 0, and $n\left( {q_{i} } \right)$ represents the number of documents containing the extended morpheme $q_{i}$. The frequency value of the term of the word list is defined by:

9 $$tf_{word} \left( {Q,d} \right) = \mathop \sum \limits_{i}^{n} IDF_{word} \left( {q_{i} ,d} \right)$$

where $n$ represents the number of expanded words in query $Q$, and $q_{i}$ represents the morpheme of each expanded word in query $Q$. The score of an expanded word in document $d$ is defined by:

10 $$Score_{word} \left( {Q,d} \right) = \frac{{tf_{word} \left( {Q,d} \right) \times \left( {k_{3} + 1} \right)}}{{tf_{word} \left( {Q,d} \right) + k_{3} \times \left( {1 - b_{2} + b_{2} \times \frac{dwl}{{avgdwl}}} \right)}}$$

where $k_{2}$ and $b_{1}$ are the adjustment factors that are usually set according to experience, and $avgdwl$ represents the average length of all word lists.

### The co-word score

**Fig. 2:**
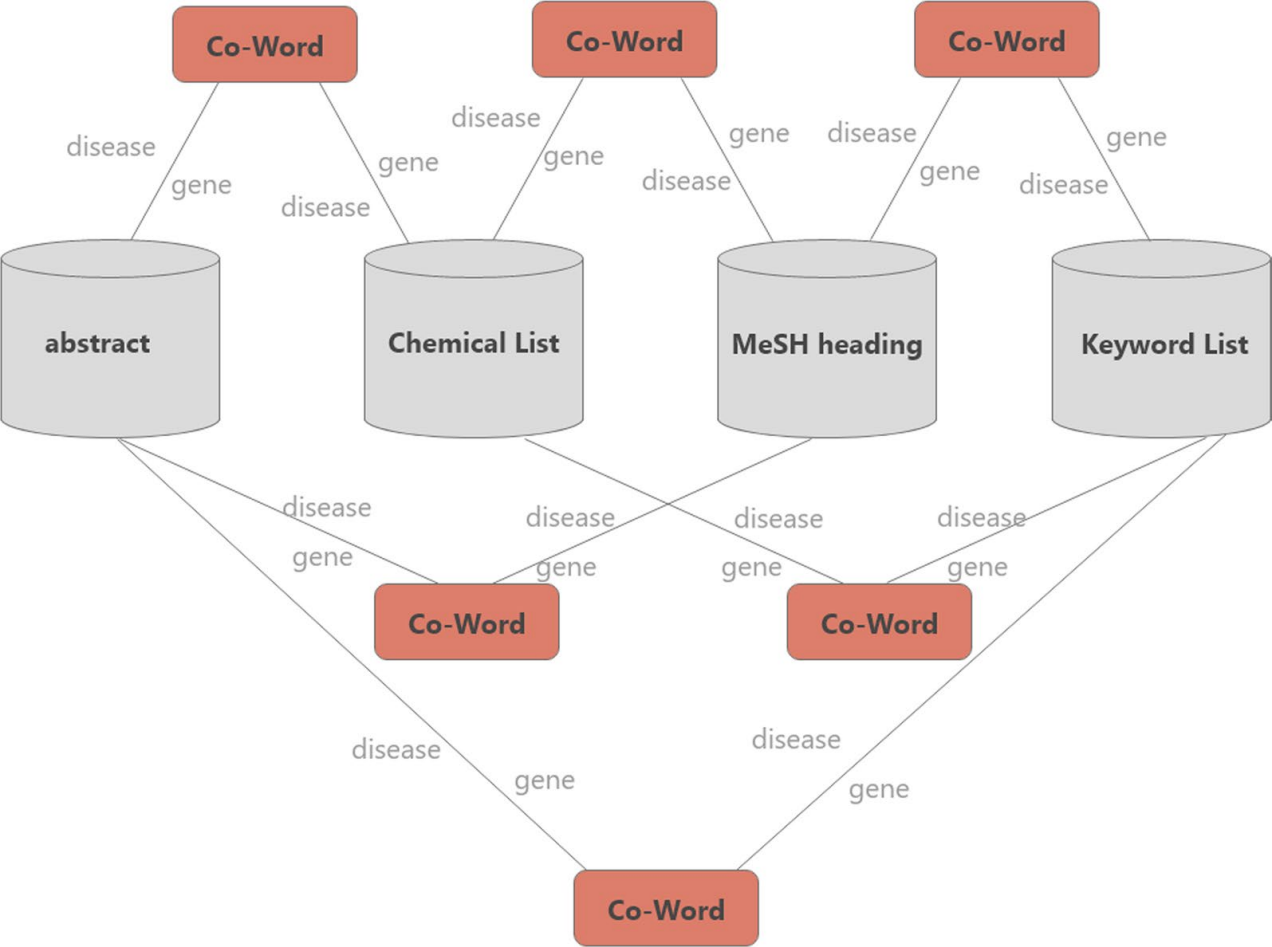
The cross co-word of abstract, chemical list, MeSH heading, and keyword list

**Fig. 3:**
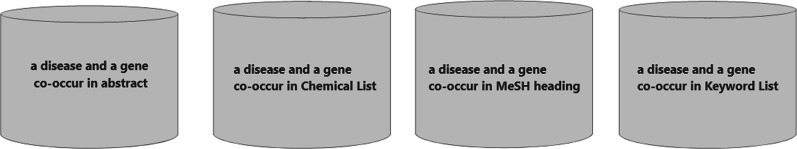
The co-word of abstract, Chemical List, Me-SH heading, and Keyword List

The co-word analysis utilizes the co-occurrence of lexical pairs or noun phrases in an article set to determine the relationship between topics in the discipline represented by the article set. In this manuscript, the co-word analysis is introduced into the article scoring model for the case when a disease and a gene co-occur across in an abstract, Chemical List, MeSH heading, and Keyword List (as presented in Fig. 2) or co-occur within any abstract, Chemical List, MeSH heading, or Keyword List (as presented in Fig. 3).

We utilize the IDF value as the co-word score to distinguish the importance of a gene, which can be formulated as:

11 $$IDF_{gene} \left( {g_{i} ,d} \right) = log\frac{{N - n\left( {g_{i} } \right) + 0.5}}{{n\left( {g_{i} } \right) + 0.5}}$$

where $N$ represents the number of documents, and $n\left( {g_{i} } \right)$ represents the number of documents containing gene morpheme $g_{i}$.

Finally, the Co-Word score is defined by:

12 $$Score_{{co}\text{-}{word}} \left( {Q,d} \right) = \mathop \sum \limits_{i}^{n} IDF_{word} \left( {g_{i} ,d} \right)$$

where $n$ is the number of genes with co-word having a disease in query $Q$, and $g_{i}$ is the morpheme of each gene in query $Q$.

### Retrieval model

We utilize the composite score as the final score for document $d$ under query $Q$, which can be formulated as:

13 $$Score_{composite} \left( {Q,d} \right) = Score_{abstract} \left( {Q,d} \right) + Score_{word} \left( {Q,d} \right) + \alpha \times Score_{{co}\text{-}{word}} \left( {Q,d} \right)$$

**Fig. 4:**
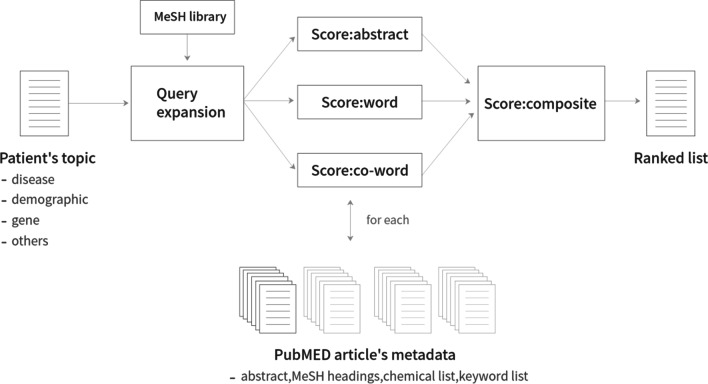
Architecture of the system retrieving biomedical articles

Figure 4 depicts the architecture of the biomedical article retrieval system.

## Parameter optimization

The proposed method has six parameters: $k_{1}$, $k_{2}$, $k_{3}$, $b_{1}$, $b_{2}$, and $\alpha$, and the choice of parameters can affect the results of information retrieval. Various algorithms, e.g., the Genetic Algorithm (GA) [25], Simulated Annealing (SA) Algorithm [26], and Ant Colony (AC) Algorithm [27], have been implemented to optimize the function in use, i.e., the objective function. With the continuous effort in developing better algorithms, several new Swarm Intelligence Optimization (SIO) Algorithms have emerged during recent years, such as Cuckoo Search (CS) [28], Glow Worm Swarm Optimization (GWSO) [29], and Particle Swarm Optimization (PSO) [30]. Among them, SIO has been widely utilized.

### Cuckoo Search Algorithm

CS is a SIO proposed by Yang et al. [28] in 2009. Guerrero et al. [31] claimed that CS outperformed GA in terms of efficiency. Some of the idealized rules utilized by CS can be given as:

1. Each cuckoo lays only one egg every time and selects a parasitic nest to randomly hatch its egg.
2. The best parasitic nest will be handed down to the next generation.
3. The number of available parasitic nests is fixed and the detection probability of parasitic nest’ master is $P_{a} \in \left( {0,1} \right)$.

The cuckoo finds the nest and updates the position according to the above-given rules. The position update formula is:

14 $$X_{i}^{{\left( {t + 1} \right)}} = X_{i}^{\left( t \right)} + T \oplus Levy\left( \lambda \right)$$

where $T$ is the step size ($T > 0$), $\oplus$ is the point-to-point multiplication operator, $Levy\left( \lambda \right)$ is the search path following the Levy distribution [32, 33]. The pseudo-code of the algorithm [28] is presented as follows:
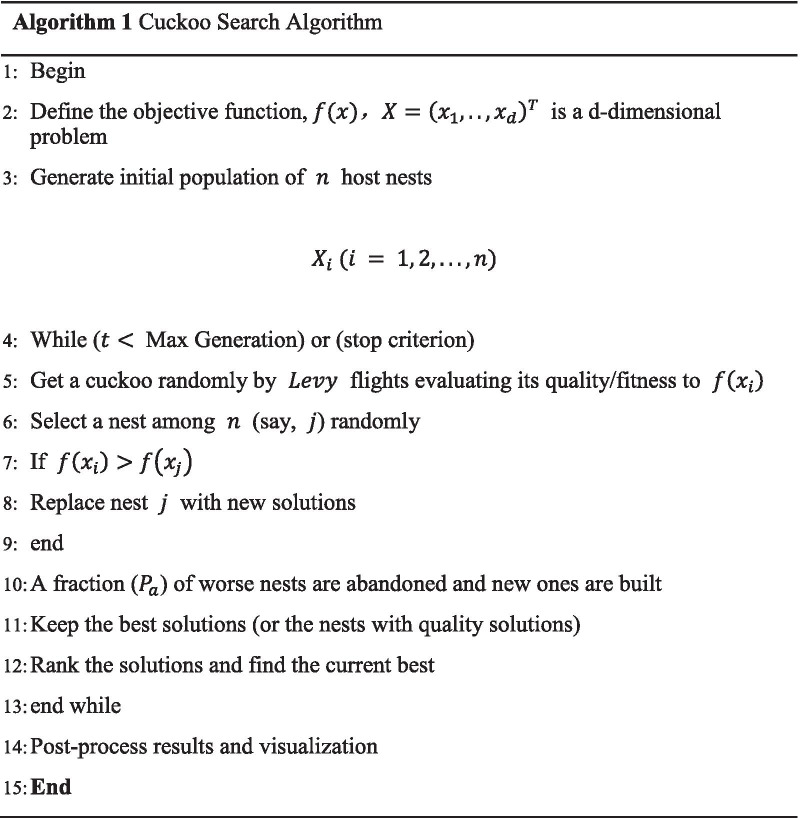


### Objective function

Precision is calculated as:

15 $$Precision = \frac{RR}{{\left( {RR + RN} \right)}}$$

where $RR$ and $RN$ refer to relevant and irrelevant documents retrieved, respectively.

Then,$P@10$ is defined as the Precision when $RR + RN = 10$. Hence, the average $P@10$ can be formulated as:

16 $$Avg_{P@10} = \frac{{\mathop \sum \nolimits_{t = 1}^{n} P@10\left( t \right)}}{n}$$

where $P@10\left( t \right)$ represents the $P@10$ value of the $t$th topic among $n$ topics.

The Normalized Discounted Cumulative Gain (nDCG) is a commonly utilized index to assess the quality of ranking in information retrieval. Let $\vartheta$ denote the relevance grade, and $gain\left( \vartheta \right)$ denote the gain associated with $\vartheta$. Also, assume that $g_{1} , g_{2} , . . . g_{z}$ are the gain values associated with the $Z$ documents retrieved by a system in response to query $q$, such that $g_{i} = gain\left( \vartheta \right)$ if the relevance grade of the document in rank $i$ is $\vartheta$. Hence, the nDCG value for this system can be calculated as:

17 $$nDCG = \frac{DCG}{{DCG_{I} }}, \quad {\text{where}}\,\, DCG = \mathop \sum \limits_{i = 1}^{Z} \frac{{g_{i} }}{{{\text{log}}\left( {i + 1} \right)}}$$

and $DCG_{I}$ denotes the $DCG$ value for an ideal ranked list for query $q$.

We define the average $nDCG$ as follows:

18 $$Avg_{nDCG} = \frac{{\mathop \sum \nolimits_{t = 1}^{n} nDCG\left( t \right)}}{n}$$

where $nDCG\left( t \right)$ represents the $nDCG$ value of the $t$ th topic among $n$ topics.

### Algorithm flow

**Fig. 5:**
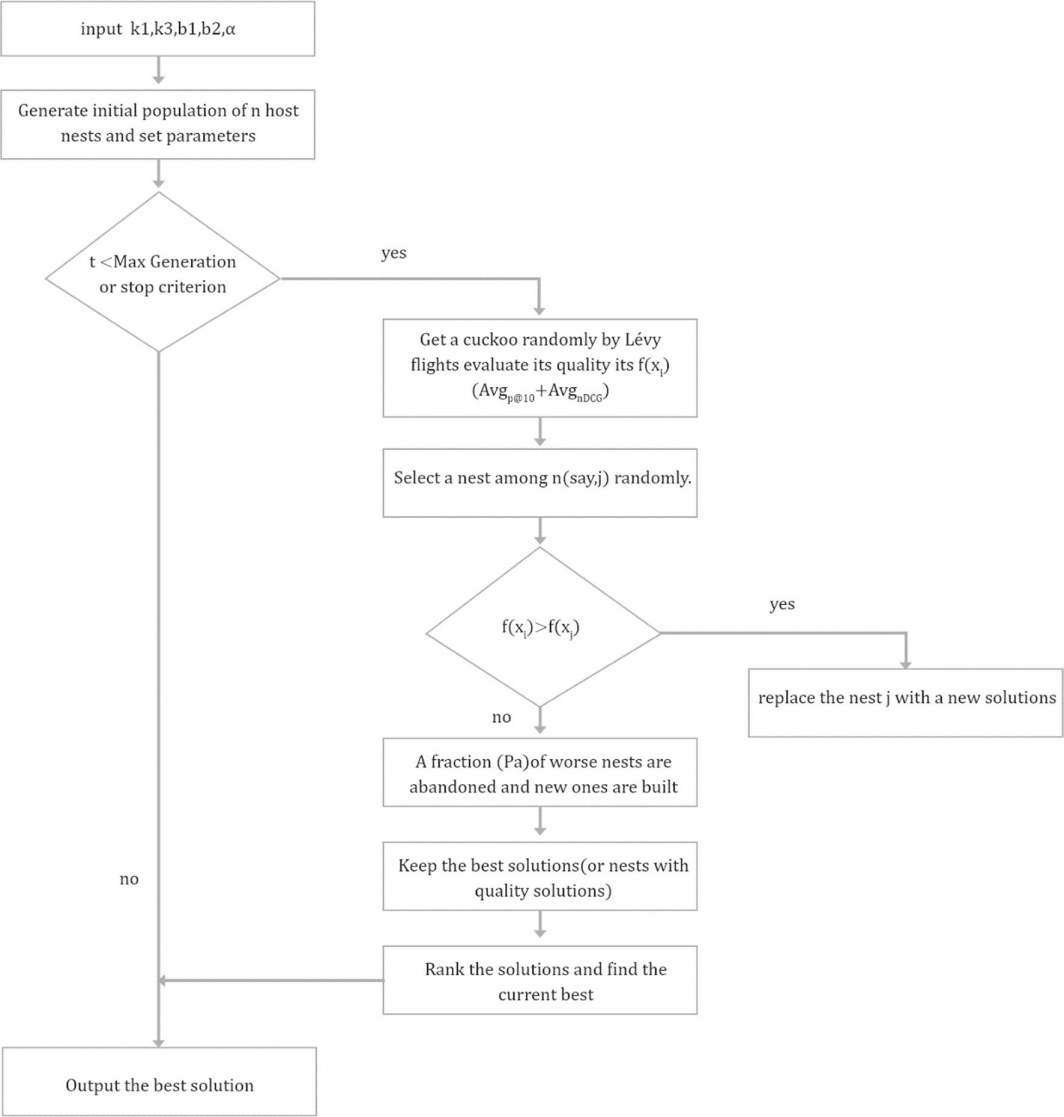
Flowchart of the proposed algorithm

Since $k_{2}$ has a fixed value ($k_{2} = 1$), we utilize $k_{1}$, $k_{3}$,$b_{1}$,$b_{2}$, and $\alpha$ as input parameters. Firstly, the algorithm generates the initial population and either set the maximum number of iterations or stop the criterion. If the number of iterations reaches the maximum number or the stop criterion is met, the algorithm ends and returns the optimal solution. Otherwise, the algorithm performs a series of operations to optimize the objective function. This manuscript defines $Avg_{P@10} + Avg_{nDCG}$ as the objective function and employs the dataset of the 2017 Precision Medicine as the training dataset to optimize the parameters. Figure 5 presents the flowchart of the proposed algorithm.

### Experimental results and comparison

#### Dataset

The data used in this work were sourced from medical articles published in 2017, 2018, and 2019 TREC Precision Medicine, which can be found on http://www.trec-cds.org/2017.html, http://www.trec-cds.org/2018.html, and http://www.trec-cds.org/2019.html, respectively. Each article was formatted using the XML 2017. The assessment results of the articles were obtained from (https://trec.nist.gov/data/precmed/qrels-final-abstracts.txt), (https://trec.nist.gov/data/precmed/qrels-treceval-abstracts-2018-v2.txt), and (https://trec.nist.gov/data/precmed/qrels-treceval-abstracts.2019.txt).

Due to the semi-structured nature of the XML format, we used MongoDB as the database for document storage and Python as the programming language. All the code can be found on the corresponding author’s GitHub (https://github.com/Bruce-V/CS-BM25).

#### Parameter setting

Table 5 presents the parameter values used in the proposed algorithm.

**Table 5:** The parameter settings of the Cuckoo Search Algorithm

Parameter	Description	Value
$n$	Population number	40
$T$	Step size	1
$Max\_Generation$	Max Generation	500
$k_{1} \_boundary$	Boundary of $k_{1}$	(0,100)
$k_{3} \_boundary$	Boundary of $k_{3}$	(0,100)
$b_{1} \_boundary$	Boundary of $b_{1}$	(0,1)
$b_{2} \_boundary$	Boundary of $b_{2}$	(0,1)
$\alpha \_boundary$	Boundary of $\alpha$	(0,5)

#### Experimental results

In Table 6, “Normal” refers to the values of empirical parameters, where CS represents the parameters trained using the 2017 dataset consisting of 1000 documents with the highest scores as a result of the selected retrieval model.

**Table 6:** The results of the Cuckoo Search Algorithm

Name	$k_{1}$	$k_{3}$	$b_{1}$	$b_{2}$	$\alpha$
“Normal”	1.2	1.2	0.75	0.75	1
CS	3.5	91.3	0.84	1	4

When the data of three years are compared, the optimized parameters are better than the empirical parameters. For an information retrieval system, the users desire related documents to appear earlier; hence, infNDCG and P@10 are two important indicators in assessing the performance of the information retrieval process. The parameters that are optimized using both NDCG and P@10 would increase the weights of the word list. The word list includes extended information about age, gender, and genes, which is crucial for distinguishing the relevant literature from the irrelevant ones. In conclusion, different parameters can be utilized to meet the needs of various users.

**Fig. 6:**
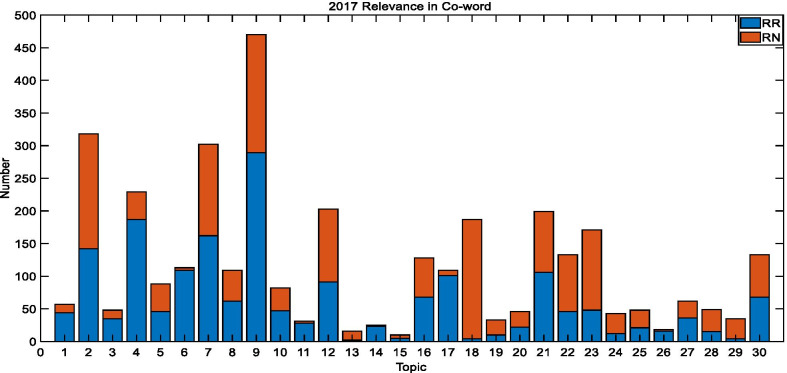
2017 TREC Precision Medicine relevance in co-word biomedical articles

**Fig. 7:**
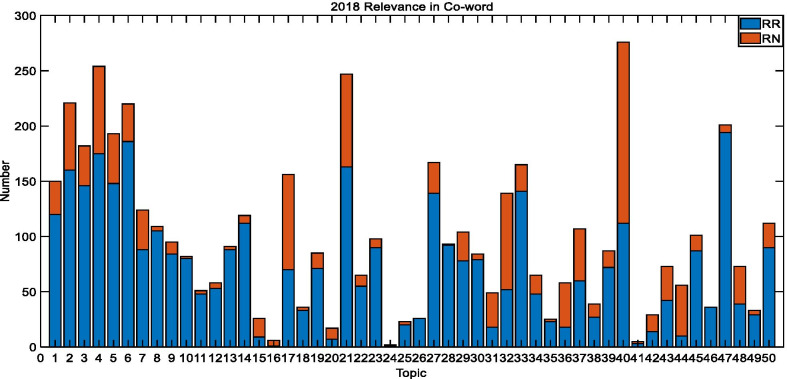
2018 TREC Precision Medicine relevance in co-word biomedical articles

**Fig. 8:**
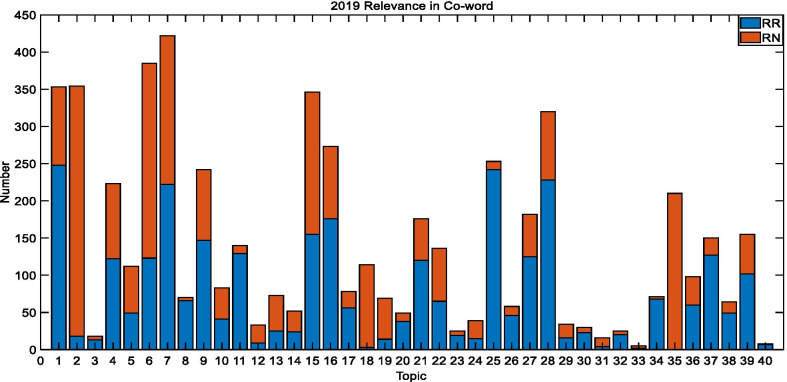
2019 TREC Precision Medicine relevance in co-word biomedical articles

In Figs. 6, 7, and 8, $RR$ represents the relevance in co-word documents, while $RN$ represents all relevance except for $RR$. It is shown that many relevant documents contain both a disease and a gene. As a result, we define the rate of average relevant document coverage as:

19 $$Avg_{cov} = \frac{{\mathop \sum \nolimits_{1}^{n} \frac{{ relevance\, in\, co{ - }word}}{ relevance }}}{n}$$

The average coverage rate of 30 topics in 2017 is 52.9%, which is 74.13% for 50 topics in 2018, and 54.4% for 40 topics in 2019. These outcomes reveal that the co-word analysis has a better impact on the retrieval of relevant documents, which greatly reduces the scope of the search.

When information retrieval is a concern, $NR$, $RR$, $NN$, and $RN$ respectively represent the relevant documents that are not retrieved, the irrelevant documents that are not retrieved, the relevant documents that are retrieved, and the irrelevant documents that are retrieved. Here, *Precision* is defined as formula (15).

*Recall* is defined as:

20 $$Recall = \frac{RR}{{\left( {RR + NR} \right)}}$$

and *F1-score* is defined as:

21 $$F1{ - }score = 2 \times \frac{Precision \times Recall}{{Precision + Recall}}$$

**Fig. 9:**
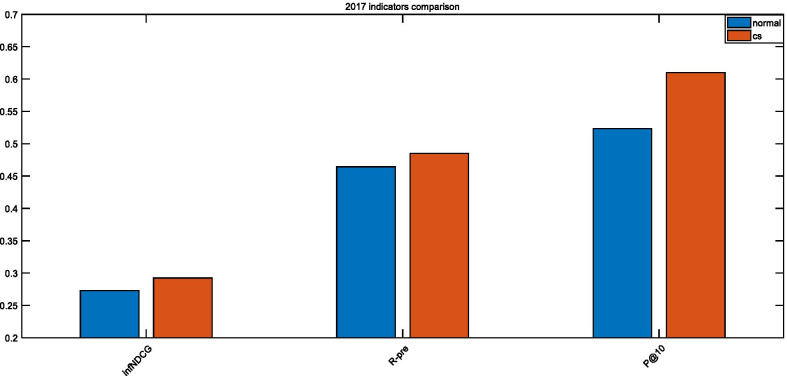
Comparison of 2017 TREC Precision Medicine indicators

**Fig. 10:**
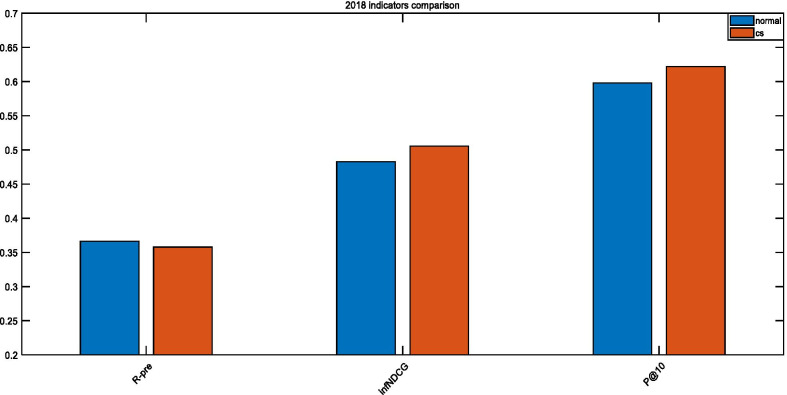
Comparison of 2018 TREC Precision Medicine indicators

**Fig. 11:**
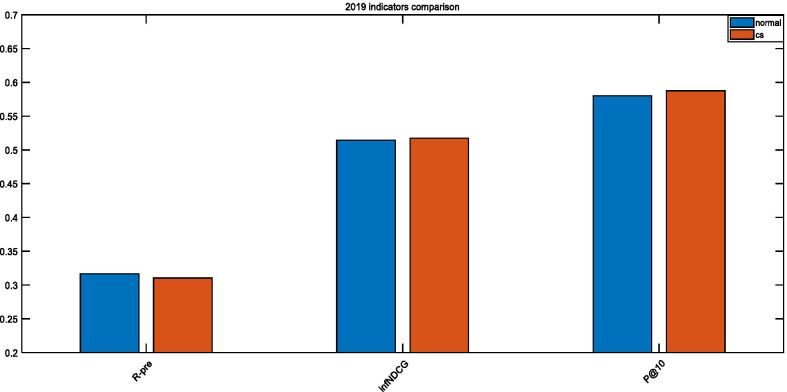
Comparison of 2019 TREC Precision Medicine indicators

As seen in Figs. 9, 10, and 11, the optimized parameters are better than the empirical parameters for both P@10 and infNDCG. Since we utilize the 2017 Precision Medicine dataset as the training set, the optimization effect is the most obvious on this dataset. When the test data in 2018 and 2019 are a concern, both P@10 and infNDCG have improved, but R-predicted has declined. This happens since the adopted objective function has improved the ranking of the most relevant documents. *Precision* and *Recall* are inversely proportional to each other when the retrieval system is a concern. However, in our case examining the retrieval of biomedical articles, we are more concerned about the precision rate to alleviate the doctors’ decision-making.

#### Experimental comparison

Considering the results of the models taken from 2017, 2018, and 2019 TREC Precision Medicine, three indicators called infNDCG, R-predicted, and P@10 are selected for comparison, and the experimental results are presented in Tables 7, 8, and 9.

**Table 7:** Experimental comparison of methods published in 2017 TREC Precision Medicine

Methods	InfNDCG	R-Prec	P@10
MayoNLPTeam [34]	0.2864	0.1698	0.3931
UCAS [35]	0.3271	0.2227	0.4276
Cbnu [36]	0.3218	0.2287	0.4614
Udel-Fang [37]	0.3879	0.2503	0.5067
Prna-Mit [38]	0.4070	0.2620	0.5300
UKNLP [39]	0.3852	0.2518	0.5533
Proposed method	0.4850	0.2924	0.6100

**Table 8:** Experimental comparison of methods published in 2018 TREC Precision Medicine

Methods	InfNDCG	R-Prec	P@10
Brown [40]	0.4000	0.2350	0.4980
Klick-Labs [41]	0.4432	0.2870	0.5400
UCAS [42]	0.5580	0.3654	0.5980
UTDHLTRI [43]	0.4797	0.2870	0.6160
Proposed Method	0.5055	0.3579	0.6220

**Table 9:** Experimental comparison of methods published in 2019 TREC Precision Medicine

Methods	InfNDCG	R-Prec	P@10
Brown [44]	0.4052	0.2527	0.4625
ECNU-ICA [45]	0.4432	0.2870	0.5400
Ims-Unipd [46]	0.4750	0.3000	0.5450
Poznan [47]	0.4800	0.3100	0.5500
Cincy-MedIR [48]	0.4801	0.3111	0.5675
CSIR-Omed [49]	0.4766	0.3165	0.5825
Proposed method	0.5172	0.3105	0.5875

We use three years of the TREC datasets to verify our experimental results. The selected methods either utilize the BM25 algorithm or its improved version. The experiments using the 2017 dataset showed a significant improvement in our proposed method for all indicators. For the 2018 dataset, our method performed better than similar algorithms for P@10, ranked second for infNDCG. The same result was observed for the 2019 dataset.

## Conclusion

This manuscript proposes a BM25-based method incorporating co-word analysis to retrieve biomedical articles. We improved the BM25 algorithm and used it to compute the score of expanded words by combining the co-word score with the gene appearance weight. Then, we utilized the Cuckoo Search Algorithm to optimize parameters on the evaluation function of both P@10 and nDCG. Optimization results suggested that increasing the score weight of the “word list” could effectively improve the ranking of the related documents. The manuscript also discusses the influence of different parameters on the retrieval algorithm and presents the parameters to meet different retrieval needs in the future. Although the proposed algorithm in this manuscript is based on the improved version of BM25, it highlights the general rules for improving the parameters of the BM25 algorithm, which were verified through numerous experiments. Since the query expansion used in this manuscript is simple, our future research will focus on adopting more linked data to investigate utilizing the topic data.

## Data Availability

The data used in this work were sourced from medical articles published in 2017, 2018, and 2019 TREC Precision Medicine, which can be found on http://www.trec-cds.org/2017.html, http://www.trec-cds.org/2018.html, and http://www.trec-cds.org/2019.html, respectively. Each article was formatted using the XML 2017. The assessment results of the articles were obtained from (https://trec.nist.gov/data/precmed/qrels-final-abstracts.txt), (https://trec.nist.gov/data/precmed/qrels-treceval-abstracts-2018-v2.txt), and (https://trec.nist.gov/data/precmed/qrels-treceval-abstracts.2019.txt). All the code can be found on the corresponding author’s GitHub (https://github.com/Bruce-V/CS-BM25).

## Abbreviations

TREC: Text Retrieval Conference
CDS: Clinical Decision Support
IR: Information Retrieval
SVM: Support Vector Machines
UMLS: Unified Medical Language System
NDCG: Normalized Discounted Cumulative Gain
GA: Genetic Algorithm
SA: Simulated Annealing
AC: Ant Colony
SIO: Swarm Intelligence Optimization
CS: Cuckoo Search
GWSO: Glow Worm Swarm Optimization
PSO: Particle Swarm Optimization
